# Contrast-medium injury of spinal cord after cerebral angiography using nonionic contrast agents: A case report and literature review

**DOI:** 10.1097/MD.0000000000036630

**Published:** 2023-12-15

**Authors:** Shitu Zhuo, Lijing Cao, Meili Yang, Jixing Chen, Zheng Yu

**Affiliations:** a Department of Neurology, The Second Affiliated Hospital of Fujian Medical University, Quanzhou, Fujian Province, China; b Translational Medicine Immunology Laboratory, Clinical Research Center, The Second Affiliated Hospital of Fujian Medical University, Quanzhou, Fujian Province, China.

**Keywords:** cerebral angiography, contrast-medium injury of spinal cord, Ioversol

## Abstract

**Introduction:**

Contrast-induced spinal cord injury (CIS) is an uncommon yet severe neurological complication following cerebral angiography. It can lead to dire consequences, including limb paralysis, respiratory distress, and even death.

**Patient concerns:**

After undergoing cerebral angiography, a 41-year-old male initially displayed symptoms of dizziness and blurred vision, which advanced into dysphoria and limb weakness within 3 hours. These initial symptoms diminished by the 12th hour. Yet, 18 hours following the procedure, the patient developed quadriplegia and paresthesia below the T5 level, even though his deep sensory functions persisted unaffected.

**Diagnosis:**

The magnetic resonance imaging and diffusion weighted imaging scans excluded the presence of cerebrovascular ischemia or subarachnoid hemorrhage. However, the magnetic resonance angiography displayed arterial vasospasms in both posterior cerebral arteries and the V4 segment of the right vertebral artery. The encephalopathy symptoms faded within 12 hours, suggesting a probable contrast-induced encephalopathy diagnosis. An magnetic resonance imaging on day 4 revealed an intensified signal in the spinal cord from C1 to T1. This finding supported the diagnosis of CIS.

**Interventions:**

Following treatment with mannitol, methylprednisolone, and nimodipine, the patient’s contrast-induced encephalopathy symptoms resolved completely within 12 hours. With a 2-week regimen of aspirin, methylprednisolone, and rehabilitative training, the neurological symptoms from CIS showed steady improvement.

**Outcomes:**

The symptoms and signs of CIS gradually improved after 2 weeks’ treatment and rehabilitation program.

**Conclusion:**

Given the grave outcomes of CIS, like limb paralysis, breathing difficulties, and even fatality, it is imperative to remain cautious about this complication, even with the use of modern, less harmful contrast agents.

## 1. Introduction

Contrast-induced injury of the spinal cord (CIS) is a rare neurological complication that occurs when intravascularly injected iodinated contrast is used during cerebral angiography. Numerous cases of CIS have been reported since the 1970s.^[1–3]^ However, the incidence of CIS has significantly decreased with the adoption of nonionic contrast agents in recent times. The most recent reported case of CIS after cerebral angiography was in 2000, where an ionic contrast medium agent was employed.^[3]^ In this article, we present a case of CIS following cerebral angiography that utilized Ioversol, a nonionic, monomeric, iodinated contrast agent, and a literature review of CIS has been proposed.

## 2. Case report

**Figure 1.**
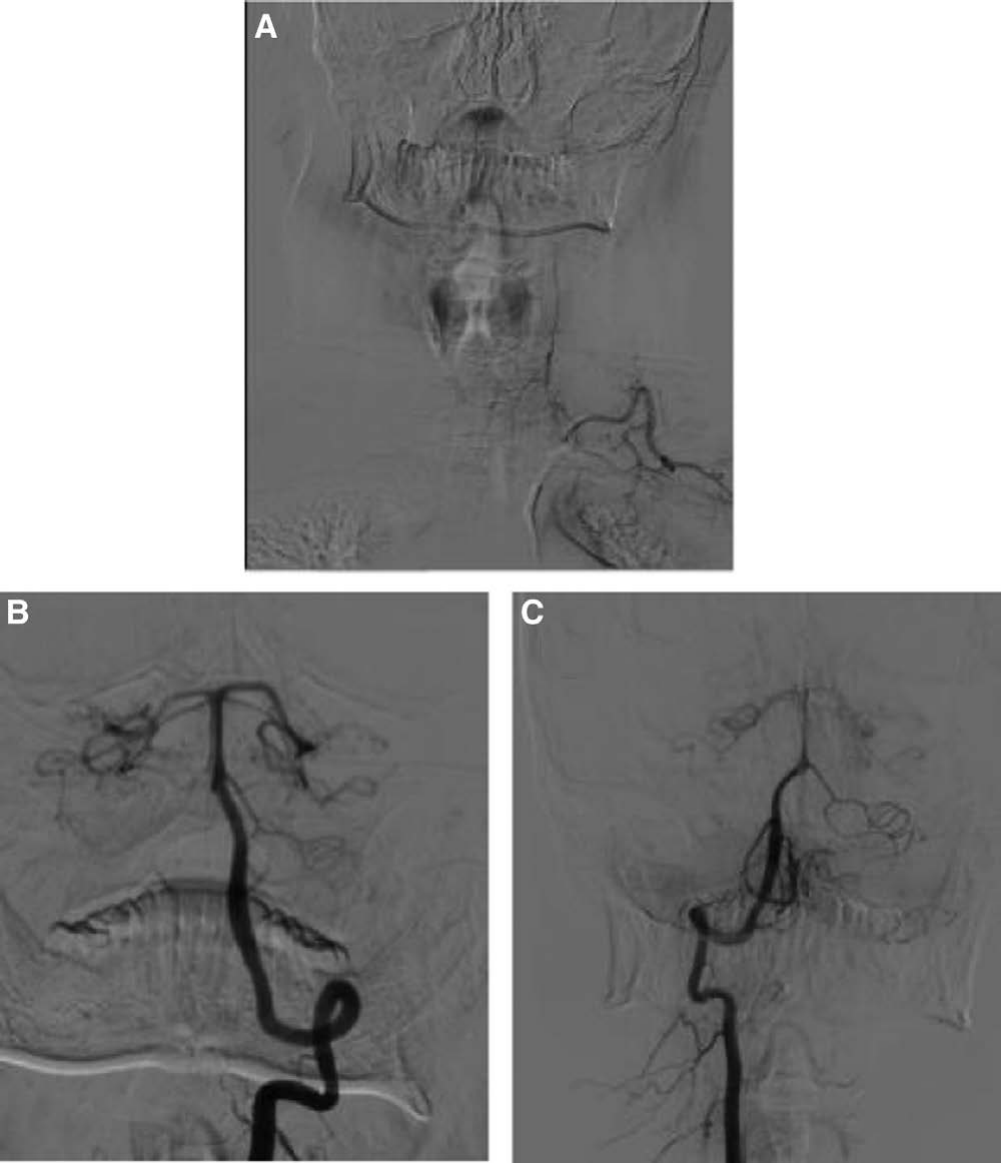
Cerebral angiography. In the initial angiogram of the left vertebral artery, the thyrocervical trunk and its branches were visualized instead of the vertebral artery (A). A follow-up angiogram of the right vertebral artery showed no abnormalities (B). A subsequent evaluation of the left vertebral artery displayed no irregularities (C).

**Figure 2.**
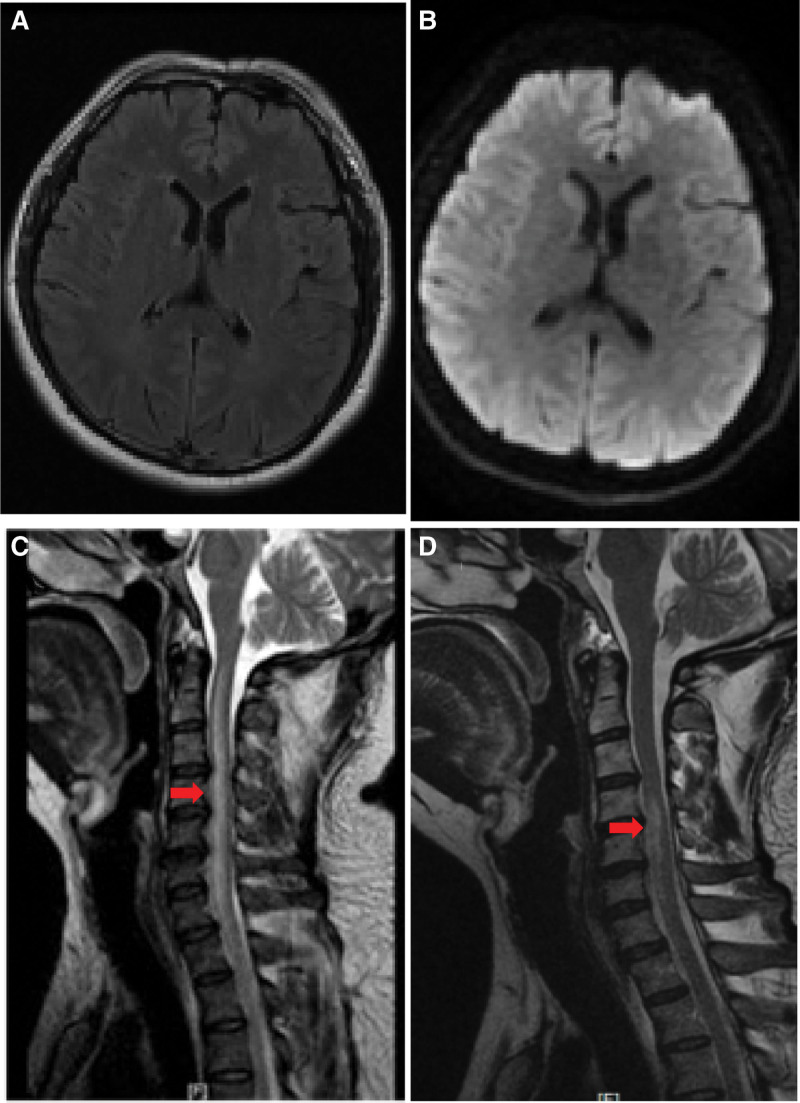
Brain, cervical, and thoracic MRI. (A) Brain FLAIR imaging 3 hours post-procedure showed no abnormalities. (B) No anomalies were detected in the DWI. (C) A lesion with elevated T2 signal intensity was identified at the anterior cervical spinal cord between C1 and T1 on hospital day 4. (D) MRI of the spinal cord on hospital week 3 demonstrated a decrease in the hyperintense region. DWI = diffusion weighted imaging, MRI = magnetic resonance imaging.

**Figure 3.**
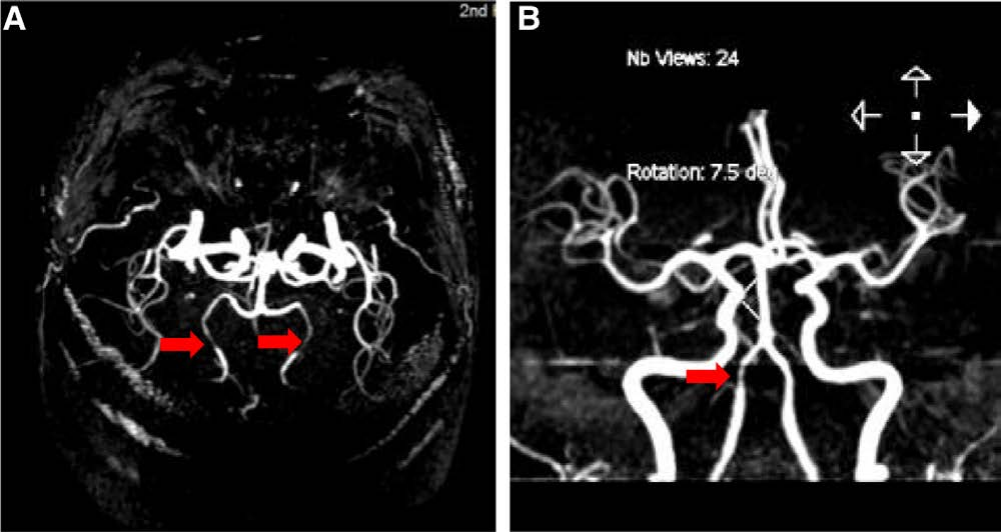
Brain MRA. (A) Bilateral posterior cerebral artery exhibited localized spasm 3 hours post-procedure. (B) Spasm in the right vertebral artery V4 segment observed at the same timeframe. MRA = magnetic resonance angiography.

A 41-year-old male with no prior medical history came in with a 2-month persistent headache. Despite regular physical and neurological evaluations indicating normal findings, computed tomography and magnetic resonance imaging (MRI) scans of the head were unremarkable. A lumbar puncture, however, displayed elevated cerebrospinal fluid pressure at 245 mm H_2_O, though other fluid analyses were normal. To investigate the possibility of cerebral venous sinus thrombosis, a cerebral angiography using the contrast agent Ioversol was performed, targeting intracranial blood vessels. Initial catheterization of the bilateral internal carotid arteries showed no vascular irregularities. When trying to visualize the left vertebral artery, a manual contrast test was done after placing the catheter near its opening. Surprisingly, during a subsequent high-pressure injection, the thyrocervical trunk and its offshoots were highlighted instead of the vertebral artery (Fig. 1A). The patient experienced a brief spell of dizziness without arm, shoulder, or neck pain during this. Despite the event, his vital signs, including blood pressure and ECG, remained stable. A subsequent angiogram of the right vertebral artery was normal (Fig. 1B). A repeat examination of the left vertebral artery was done to exclude potential complications, revealing no abnormalities (Fig. 1C). The procedure concluded without further incidents, lasting 34 minutes and using 90 mL of contrast. Three hours post-procedure, the patient experienced exacerbated headaches, blurred vision, dysphoria, and a significant reduction in vision, only sensing light. Brain MRI and diffusion weighted imaging scans ruled out acute ischemic stroke (Fig. 2A and B) or subarachnoid hemorrhage (SAH). However, magnetic resonance angiography (MRA) displayed spasms in both posterior cerebral arteries (Fig. 3A) and the right vertebral artery’s V4 segment (Fig. 3B), pointing to a potential diagnosis of contrast-induced encephalopathy (CIE).^[4,5]^ After treatment with mannitol, methylprednisolone, and nimodipine, the patient’s symptoms fully subsided within 12 hours. But, by the following day (18 hours after the surgery), he exhibited paralysis in his limbs and reduced sensory perceptions below the T5 level, though proprioception and vibration senses were intact. A cervical MRI on day 4 showcased a high-intensity signal on the T2 sequence between C1 and T1 (Fig. 2C and D), suggestive of an anterior spinal artery syndrome. This led to a diagnosis of CIS. After 2 weeks of treatment with aspirin, methylprednisolone, and rehabilitation training, the patient’s neurological impairment progressively improved. A follow-up MRI on week 3 indicated the spinal hyperintensity had regressed. By week 4, the patient was discharged, fully mobile, and self-sufficient.

## 3. Discussion

Cerebral angiography is associated with rare neurological complications, such as CIE and CIS. Although CIE cases have been frequently reported over the past 2 decades cortex,^[6,7]^ CIS occurrences have been relatively scarce.^[4,8]^ Here, we present a unique case where both CIE and CIS manifested concurrently after cerebral angiography using Ioversol. Shortly after the cerebral angiography procedure, the patient exhibited dizziness and visual blurring, followed by dysphoria and limb weakness 3 hours later. Brain MRI and diffusion weighted imaging ruled out cerebrovascular ischemia or SAH (Fig. 2A and B). MRA revealed arterial vasospasm in the bilateral posterior cerebral arteries (Fig. 3A) and the right vertebral artery V4 segment (Fig. 3B). All encephalopathy symptoms resolved within 12 hours. However, the next day (18 hours after the surgery), quadriplegia, and paresthesia below the T5 level, with preserved deep sensory function, became evident. MRI on the fourth day indicated a hyperintense area in the spinal cord opposite C1-T1 (Fig. 2C and B), indicative of anterior spinal artery syndrome and supporting the diagnosis of CIS. This case highlights the occurrence of both CIE and CIS following cerebral angiography with nonionic contrast agents. Despite their rarity, healthcare professionals should remain vigilant about the possibility of CIS, given its potential to cause substantial disability.

## 4. Clinical character

Since the 1970s, a total of 9 cases of CIS have been reported, including the case presented in this study (Table 1) in 21st century.^[1–3,9–12]^ Notably, males exhibit a higher susceptibility to CIS following cerebral angiography, with a male-to-female ratio of 7:2. Furthermore, all affected patients were young or middle-aged individuals. Angiography procedures were performed for various reasons: SAH in 3 cases, intracranial hypertension in 2 cases, space-occupying lesions in 1 case, intermittent diplopia in 1 case, and epilepsy in 1 case. It is worth noting that CIS does not manifest in ischemic cerebrovascular diseases, whereas CIE can be observed in these conditions. Of the patients studied, 3 underwent vertebral angiography and 6 had transfemoral angiography. None had transradial angiography. The onset of all CIS occurred within 24 hours after angiography. Among the 9 patients, except for 1 case of left hemiparesis, the remaining 8 experienced quadriplegia and paresthesia, indicating that the disease affected the high cervical segment. Furthermore, there were no symptoms of posterior myelopathy, suggesting that the lesions were confined to the anterior spinal artery area. The dose of contrast medium used during the angiography procedures ranged from 5 mL to 190 mL, implying that there was no direct relationship between the occurrence of CIS and the dose of contrast medium. The prognosis was severe in 5 cases and good in 3 cases.

**Table 1:** Clinical characteristics of 9 cases of contrast-induced spinal cord injury.

Time	Authors	Gender	Age	Medical reasons	Past medical history	Approach	Onset time	Clinical symptoms	Total amount	Contrast agent	Treatment	Outcome
1962	A.Ederli^[9]^	Male	54	Intracranial hypertension	Suspected neoplasm	Vertebral angiography	Immediately	Quadriplegia	30 mL	Triode 2, 4, 6 acetyl-amino-3-benzoate	NA	Died
1968	Howieson J^[10]^	Male	23	Occupying lesions	NA	Vertebral angiography	Immediately	Weakness numbness (upper limbs)	NA	NA	NA	Recovered
1968	Howieson J^[10]^	Male	46	Subarachnoid hemorrhage	Without	Vertebral angiography	Immediately	Paresthesia, quadriplegia	190 mL	Meglumine iothalamate	Vasopressor agents, LMD	quadriplegic
1969	Mutsumasa Takahashi^[11]^	Male	15	NA	NA	Femoral catheter	NA	Quadriplegia	12 mL	60% methylglucamine iothalamate	NA	Quadriplegia
1971	LYNN W.	Female	47	Subarachnoid hemorrhage	NA	Percutaneous transfemoral approach.	During operation	Paresthesia, quadriplegia	45 mL	Renografin-60	NA	able to walk with assistance
1977	Ramirez-Lassepas^[2]^	Male	47	Intermittent diplopia	NA	Femoral catheter	4 min	Paresthesia, quadriplegia	8 mL	Renografin-60	Dexamethasone, LMD	NA
1998	G.K.Bejjani^[1]^	Female	54	Subarachnoid hemorrhage	Hypertension	Seldinger technique	45 min	Hemiparesis(L)	150 mL	Hexabrix and Conray 60	Rehabilitation, decompressive, laminectomy	good
2000	Mayank Pathak^[3]^	Male	46	Epilepsy?	NA	Transfemoral catheterization	Immediately	Paralyzed below C4	5 mL	Sodium diatrizoate	NA	Paraplegia
2023	Shitu Zhuo	Male	41	Intracranial hypertension	Without	Femoral catheter	18 hours	Paresthesia, quadriplegia	90 mL	Ioversol	Aspirin, methylprednisolone, and rehabilitation	Walk independently

## 5. Pathogenesis mechanisms

The pathogenesis mechanisms underlying CIS remain uncertain. Toxicological effects of drugs may play a direct role, including the release of endothelin induced by contrast media, which leads to blood-brain barrier breakdown. This, in combination with chemotoxic effects from contrast hyperosmolarity and direct neurotoxicity, can induce arterial vasospasm and tissue edema.^[13]^ Animal and postmortem studies have provided insights into the effects of contrast media hyperosmolarity, which can draw water out of the endothelial cells of vessels, resulting in the shrinking of intracellular space, widening of gap junctions, and an increase in the permeability of the blood-brain barrier.^[14]^ Furthermore, a co-occurrence of direct toxic action of contrast media and ischemic injury has been observed.^[15]^

Vasospasm is widely recognized as a pathophysiological outcome of both CIE and CIS, often attributed to the direct toxicity of contrast agents. Furthermore, it is also postulated as a primary pathogenic mechanism underlying these conditions. The application of arterial spin labeling MRI perfusion imaging in 2019 enabled the identification of cerebral hypoperfusion resulting from arterial vasospasm in cases of CIE.^[16]^ This innovation provided empirical evidence supporting the role of vasospasm in the pathogenesis of CIE. In the recent case we presented, MRA pinpointed focal constriction in both cerebral and vertebral arteries during the CIE phase, prior to the onset of CIS. This observation bolsters the assertion that vasospasm serves as a fundamental pathogenic mechanism in CIS.

It is important to note that further research is needed to fully understand the intricate pathogenesis processes underlying CIS. Toxic effects of agent, and the complex interactions between contrast media and cerebral tissues requires further investigation to improve our knowledge and preventive measures for this condition.

## 6. Risk factors

Several specific risk factors have been reported in CIS cases, including the concurrent presence of cervical spondylosis, accidental injection into the left thyrocervical trunk, using ionic monomer contrast media, an uncomfortable head position due to taping, and higher sedative intake.^[1,3,9]^

In a study by K. Bejjani, a patient with CIS after cerebrovascular angiography was found to have cervical spondylosis with spinal stenosis, which was suggested as a risk factor for CIS. The direct compression of the spinal cord caused by a slipped disc and/or resulting ischemia may contribute to the onset of CIS in patients with cervical spondylosis.^[1]^ In our case, MRI findings revealed cervical spondylosis at the C3-4, C4-5, C5-6, and C6-7 levels with canal stenosis, which may contribute to the development of CIS in this particular case.

The left vertebral artery is often the dominant supplier of blood to the upper and middle cervical cord in many individuals. Only the highest cervical segments receive blood supply from branches originating from the intracranial parts of the vertebral arteries, which constitute the most rostral portion of the anterior spinal artery. As the anterior spinal artery descends, it merges with the fields of the upper 2 to 3 cervical arteries, with dominance on the left side. These upper cervical arteries anastomose with and contribute to the filling of the anterior spinal artery in this region. In certain cases, only one functional cervical radicular artery may be present, originating either from the vertebral artery (mainly at mid-cervical levels) or from the ascending cervical branch of the thyrocervical trunk (at mid-to lower cervical levels). This anatomical arrangement likely accounts for the cervical spinal cord necrosis that followed the unintended injection into the left thyrocervical trunk during an attempted vertebral artery catheterization. In our case, an accidental injection into the left thyrocervical trunk occurred, which could be a risk factor for CIS. Mistakenly injecting into the left thyrocervical trunk might compromise blood flow to the cervical cord, leading to the devastating consequence of spinal cord necrosis.

Ionic monomer contrast media, due to its hyperosmolality and direct neurotoxicity,^[17]^ is more likely to cause CIE and CIS. Until this report, there were no known instances of CIS resulting from the use of nonionic contrast agents, which boast reduced osmolality and neurotoxicity. Their lower osmolality has made them safer, decreasing neurotoxic risks linked to contrast media. Therefore, adopting nonionic contrast agents in medical practices could significantly reduce the incidence of CIS.

## 7. Conclusion

We reported a unique case of CIS post CIE after cerebral angiography, marking the first reported instance of CIS triggered by nonionic contrast media. The underlying cause may be vertebral artery vasospasm. Potential risk factors include cervical spondylosis with canal stenosis and an unintended injection into the left thyrocervical trunk. Given the grave outcomes of CIS, like limb paralysis, breathing difficulties, and even fatality,^[1,3]^ it is imperative to remain cautious about this complication, even with the use of modern, less harmful contrast agents.

## Acknowledgments

The authors extend their deepest gratitude to the patient. His willingness to provide informed consent for the publication of this case has been instrumental in furthering our understanding and sharing knowledge in this field.

## Author contributions

**Conceptualization:** Meili Yang.

**Data curation:** Lijing Cao, Jixing Chen.

**Formal analysis:** Lijing Cao.

**Investigation:** Jixing Chen.

**Writing – original draft:** Shitu Zhuo, Lijing Cao.

**Writing – review & editing:** Zheng Yu.
